# Elimination of Aicardi–Goutières syndrome protein SAMHD1 activates cellular innate immunity and suppresses SARS-CoV-2 replication

**DOI:** 10.1016/j.jbc.2022.101635

**Published:** 2022-01-25

**Authors:** Adrian Oo, Keivan Zandi, Caitlin Shepard, Leda C. Bassit, Katie Musall, Shu Ling Goh, Young-Jae Cho, Dong-Hyun Kim, Raymond F. Schinazi, Baek Kim

**Affiliations:** 1 Department of Pediatrics, School of Medicine, Emory University, Atlanta, Georgia, USA; 2 Department of Pharmacy, College of Pharmacy, Kyung-Hee University, Seoul, South Korea; 3 Center for Drug Discovery, Children’s Healthcare of Atlanta, Atlanta, Georgia, USA

**Keywords:** SAMHD1, SARS-CoV-2, innate immunity, JAK pathway, Stat1, ACE2, angiotensin-converting enzyme 2, AGS, Aicardi–Goutières syndrome, ARHGEF5, Rho guanine nucleotide exchange factor 5, cDNA, complementary DNA, COVID-19, coronavirus disease 2019, DMEM, Dulbecco's modified Eagle's medium, FBS, fetal bovine serum, FDA, US Food and Drug Administration, HCoV-OC43, human coronavirus OC43, IFN, interferon, IRF3, IFN regulatory factor 3, ISG, IFN-stimulated gene, JAK, Janus kinase, MDM, monocyte-derived macrophage, MOI, multiplicity of infection, NSP, nonstructural protein, pSTAT1, phosphorylated form of STAT1, qRT–PCR, quantitative RT–PCR, SAMHD1, sterile alpha motif and histidine–aspartate domain–containing protein 1, SARS-CoV-2, severe acute respiratory syndrome coronavirus 2, STAT1, signal transducer and activator of transcription 1, VLP, virus-like particle, Vpx, viral protein X

## Abstract

The lack of antiviral innate immune responses during severe acute respiratory syndrome coronavirus 2 (SARS-CoV-2) infections is characterized by limited production of interferons (IFNs). One protein associated with Aicardi–Goutières syndrome, SAMHD1, has been shown to negatively regulate the IFN-1 signaling pathway. However, it is unclear whether elevated IFN signaling associated with genetic loss of *SAMHD1* would affect SARS-CoV-2 replication. In this study, we established *in vitro* tissue culture model systems for SARS-CoV-2 and human coronavirus OC43 infections in which SAMHD1 protein expression was absent as a result of CRISPR–Cas9 gene KO or lentiviral viral protein X–mediated proteosomal degradation. We show that both SARS-CoV-2 and human coronavirus OC43 replications were suppressed in SAMHD1 KO 293T and differentiated THP-1 macrophage cell lines. Similarly, when SAMHD1 was degraded by virus-like particles in primary monocyte-derived macrophages, we observed lower levels of SARS-CoV-2 RNA. The loss of SAMHD1 in 293T and differentiated THP-1 cells resulted in upregulated gene expression of IFNs and innate immunity signaling proteins from several pathways, with STAT1 mRNA being the most prominently elevated ones. Furthermore, SARS-CoV-2 replication was significantly increased in both SAMHD1 WT and KO cells when expression and phosphorylation of STAT1 were downregulated by JAK inhibitor baricitinib, which over-rode the activated antiviral innate immunity in the KO cells. This further validates baricitinib as a treatment of SARS-CoV-2–infected patients primarily at the postviral clearance stage. Overall, our tissue culture model systems demonstrated that the elevated innate immune response and IFN activation upon genetic loss of *SAMHD1* effectively suppresses SARS-CoV-2 replication.

Human severe acute respiratory syndrome coronavirus 2 (SARS-CoV-2), which is the causative agent of the current coronavirus disease 2019 (COVID-19) pandemic, harbors strong potential for massive and disruptive inflammatory responses in infected individuals (1, 2). While interferons (IFNs) are crucial as the first line of host defense against invading pathogens, especially during virus infections, coronaviruses have been previously reported as weak IFN inducers. During SARS-CoV-1 infections, the vital antiviral activities of host IFNs are crucially absent as a result of IFN regulatory factor 3 (IRF3) phosphorylation inhibition by the viral papain-like protease (3). Infections with other beta-coronaviruses or alpha-coronaviruses such as the Middle East Respiratory Syndrome coronavirus (4) and porcine epidemic diarrhea virus (5) were also demonstrated to induce weak IFN responses. In general, SARS-CoV-2 infections have been characterized by an imbalance between excessive production of proinflammatory cytokines and limited levels of IFN-1 in patients (2). However, depending on the cell types tested, there have been contrasting findings on the stimulatory effects of SARS-CoV-2 infections on IFN signaling thus far. Signal transducer and activator of transcription 1 (STAT1) as well as its activated phosphorylated form (pSTAT1) was upregulated following SARS-CoV-2 infection in Calu-3 cells, but infected Vero and polarized human airway epithelial cells exhibited lower pSTAT1 expression than that of mock-infected cells (6). As demonstrated by a more recent study, cell lysates collected from SARS-CoV-2-infected Calu-3 cells showed that STAT1 remained phosphorylated for a longer period up to 72 h postinfection, whereas pSTAT1 could only be detected in Vero cells up to 24 h (7). In a separate report, different components of the Janus kinase (JAK)–STAT pathway, namely JAK1, tyrosine kinase 2, and IFN alpha receptor subunit 1, were suppressed following SARS-CoV-2 infections in various human cell lines, including human-induced pluripotent stem cell–derived cardiomyocytes (8).

Nonetheless, it is clear that the expression levels and timing of induced antiviral IFN responses are crucial for SARS-CoV-2 prognosis and disease progression. It has been reported that IFNs produced following SARS-CoV-2 infection were insufficient to suppress viral replication in Calu-3 and A549–angiotensin-converting enzyme 2 (ACE2) cell lines (9). In fact, exogenous IFN was only effective in downregulating SARS-CoV-2 replication if the cells were pretreated prior to virus exposure, whereas postinfection treatment was rendered ineffective (9). Another study has also suggested that the activity of IFN-stimulated gene (ISG)–mediated host defense during the initial infection of SARS-CoV-2 harbors crucial impact on the subsequent virus replication in infected cells (10). Prior infection with rhinovirus, which resulted in elevated IFN and ISG expression, effectively inhibited SARS-CoV-2 replication in human airway epithelial organoids. Hence, it should be highlighted that while insufficient IFN responses were generally reported in severe COVID-19 cases, the important protective effects of IFN among mild or asymptomatic patients should not be neglected. The presence of autoantibodies against IFN-1 in critically symptomatic COVID-19 patients correlated with higher nasopharyngeal SARS-CoV-2 copy numbers than that of individuals who exhibit mild symptoms and do not express the autoantibodies (11). An analysis of immune cells isolated from mild and severe COVID-19 patients demonstrated that STAT1 phosphorylation and IRF9 expression, which were elevated in mild symptomatic patients, were suppressed in critical stage individuals harboring higher viral load (12). Numerous anti-SARS-CoV-2 therapeutic approaches are being extensively explored both preclinically and clinically. Among them, treatments with IFNs are generating promising outcomes, particularly in early stages of infection (13, 14), supporting that IFNs and IFN responses can serve as key antiviral tools for early therapeutic COVID-19 interventions. More specifically, lower viral titers were detected in Calu-3 and Vero E6 cells when exposed to IFN-1 16 h prior to SARS-CoV-2 infection (15). Viral replication was significantly enhanced in IFN-competent Calu-3 cells when JAK–STAT signaling inhibitor, ruxolitinib, was added, further suggesting that SARS-CoV-2 replication is sensitive to IFN-1 antiviral activities. Another publication also reported that IFN-β-1a treatment following the infection of SARS-CoV-2 effectively inhibited viral replication in Vero E6 cells (EC_50_ = 1.971–4.682 IU/ml) (13).

Concurrent with these studies, various clinical trials have been conducted in response to the urgent need for an effective anti-SARS-CoV-2 therapeutic option in the event of the continuous, yet rapid rise of new COVID-19 cases around the globe. In a clinical trial comprising of 127 patients, a combination therapy consisting of ribavirin, lopinavir–ritonavir, and IFN-β-1b was able to alleviate symptoms as well as reduce viral load and duration of hospital stay among treated patients relative to the control group (16). A separate study involving 33 COVID-19 patients administered with aerosol inhalation of IFN-kappa and the immune modulator, trefoil factor 2, showed improvements in disease progression and reduction in viral load (17). Recently, the US Food and Drug Administration (FDA) has approved the use of another JAK–STAT signaling inhibitor, baricitinib, as a treatment option for hospitalized and pediatric COVID-19 patients who require supplemental oxygen support (https://www.fda.gov/media/143822/download). COVID-19 patients who have been subjected to a short-course treatment of baricitinib in combination with the antimalarial drug, hydroxychloroquine, exhibit improved clinical outcomes (18). Separate reports from multinational double-blind and placebo-controlled clinical trials (the Adaptive COVID-19 Treatment Trial 2 and COV-BARRIER) also demonstrated that baricitinib treatments contributed to improved disease progressions and recovery rates among COVID-19 patients who were provided with the standard of care treatments including remdesivir or dexamethasone (19, 20).

A series of human genetic diseases induce innate immunity activation. In particular, Aicardi–Goutières syndrome (AGS) is a neuroimmunological genetic disorder that induces constant activation of innate immunity even in the absence of any pathogen infections, leading to neurodevelopmental complications and early death (21, 22, 23). The immunological hallmark of AGS is hyperactivation of IFN-1 responses and excessive production of IFN-α (21, 24). Sterile alpha motif and histidine–aspartate domain–containing protein 1 (SAMHD1) is one of the known genes responsible for AGS, including *RNaseH2*, *Trex1*, *ADAR*, and *IFIH1* (25, 26, 27, 28, 29). SAMHD1 protein is a dNTP triphosphohydrolase that depletes cellular dNTPs, which if disrupted, can induce failure of nucleic acid metabolism and activate nucleic acid sensing mechanisms (30, 31, 32, 33). SAMHD1 also suppresses the cellular innate immune response by interacting with proteins involved in the NF-B and IFN-1 pathways (34). Conversely, as observed in AGS patients, genetic loss of *SAMHD1* results in failure of negative regulation of the IFN responses (27).

In this study, we generated *in vitro* tissue culture systems that enabled us to investigate the outcomes of the genetic loss of *SAMHD1* on coronavirus (SARS-CoV-2 and human coronavirus OC43 [HCoV-OC43]) replication. Overall, our findings support that the activation of the innate immunity and IFN responses regulated by the AGS protein, SAMHD1, can effectively suppress both SARS-CoV-2 and HCoV-OC43 replication.

## Results

### SAMHD1 KO in 293T cells inhibit SARS-CoV-2 and HCoV-OC43 replication

**Figure 1:**
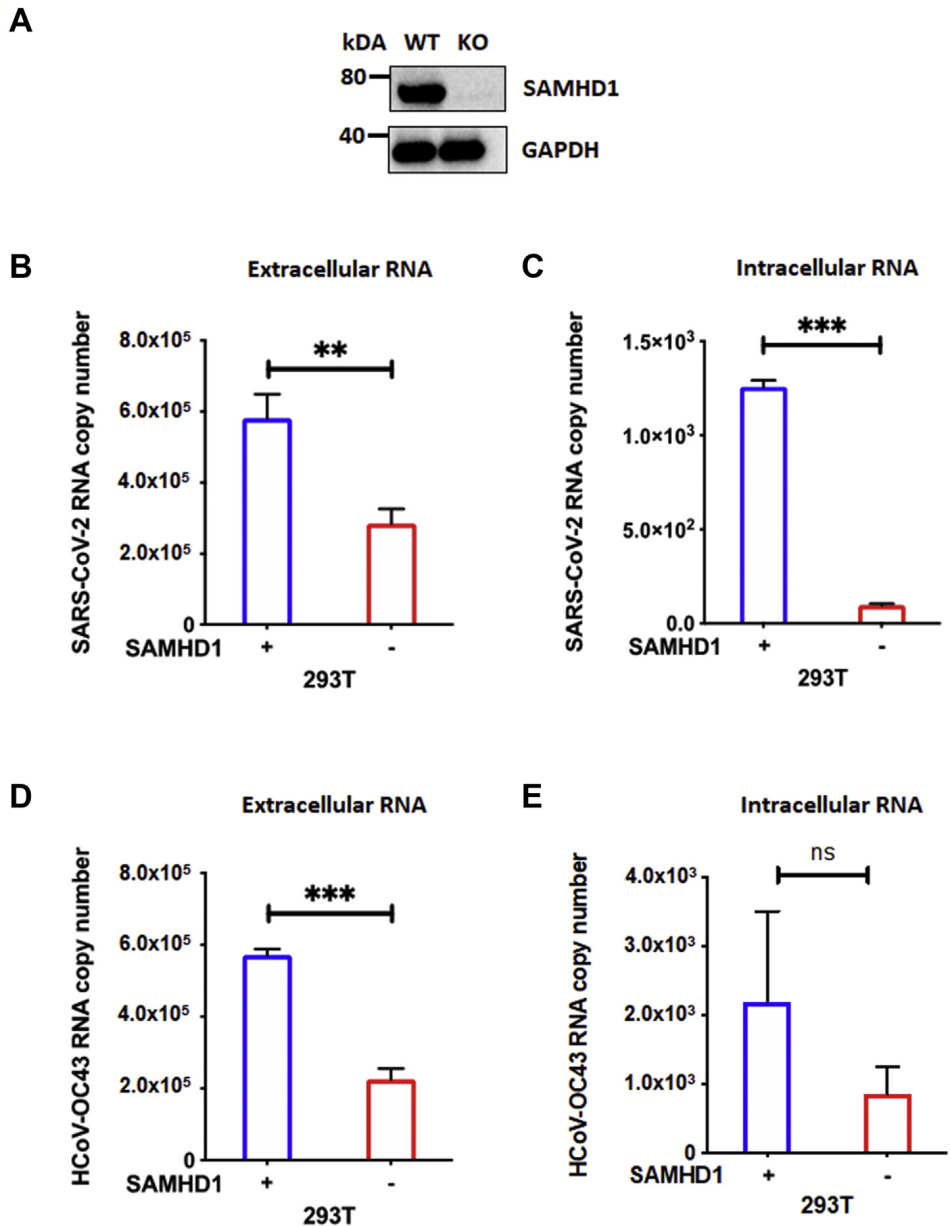
**SAMHD1 loss suppresses SARS-CoV-2 and HCoV-OC43 replication in 293T cells.***A*, SAMHD1 expression in SAMHD1 WT and KO 293T cells was confirmed *via* Western blot using antihuman SAMHD1 antibody. GAPDH was used as a loading control. Separately, using 96-well plates, SAMHD1 WT (*blue*) and KO (*red*) 293T cells were infected with either (*B* and *C*) SARS CoV-2 or (*D* and *E*) HCoV-OC43 at MOI 0.1 in triplicates. Extracellular (*B* and *D*) and intracellular (*C* and *E*) RNAs were isolated from collected media and harvested cells, respectively, on day 2 postinfection for qRT–PCR analyses. The data are presented as means of triplicates, and the standard deviations from the means are represented as error bars. HCoV-OC43, human coronavirus OC43; MOI, multiplicity of infection; qRT, quantitative RT; SAMHD1, sterile alpha motif and histidine–aspartate domain–containing protein 1; SARS-CoV-2, severe acute respiratory syndrome coronavirus 2.

SAMHD1 displays either a proviral effect or an antiviral effect, depending on the type of virus studied. While SAMHD1 restricts HIV-1 (30, 31, 35) and Herpes simplex virus (36) in nondividing macrophages *via* its dNTPase activity, Zika virus and Chikungunya virus have been reported to benefit from its suppressive effects on host antiviral innate immunity (37). Here, we first tested the role of SAMHD1 in the replication of SARS-CoV-2 and another beta-coronavirus, HCoV-OC43. For this test, we employed the CRISPR–Cas9 method to KO *SAMHD1* gene in 293T cells (SAMHD1 KO) as this cell line is significantly susceptible to SARS-CoV-2 and supports productive viral replication (38). SAMHD1 expression in SAMHD1 WT and KO 293T cells were confirmed using Western blot (Fig. 1*A*). SAMHD1 WT and KO 293T cells seeded in triplicates in 96-well plates were infected with SARS-CoV-2 (multiplicity of infection [MOI] = 0.1) or HCoV-OC43 (MOI = 0.1). Intracellular RNAs from the harvested cells and extracellular RNAs from the collected media were isolated on day 2 postinfection. As shown in Figure 1, *B*–*E*, we detected significantly higher extracellular SARS-CoV-2 and HCoV-OC43 viral yield in the media relative to the intracellular RNA samples, hence indicating that both viruses were productively replicating and released into the extracellular environment in 293T cells. As shown in Figure 1, *B* and *C*, both extracellular and intracellular SARS-CoV-2 RNA copy numbers were significantly reduced in SAMHD1 KO 293T cells (*red*) compared with WT 293T cells (*blue*). A similar pattern was also observed with HCoV-OC43 (Fig. 1, *D* and *E*). Overall, the data in Figure 1 demonstrate that the loss of SAMHD1 expression leads to the suppression of SARS-CoV-2 and HCoV-OC43 replication in 293T cells.

### SAMHD1 KO in differentiated THP-1 macrophages induce the reduction of intracellular SARS-CoV-2 and HCoV-OC43 RNA levels

**Figure 2:**
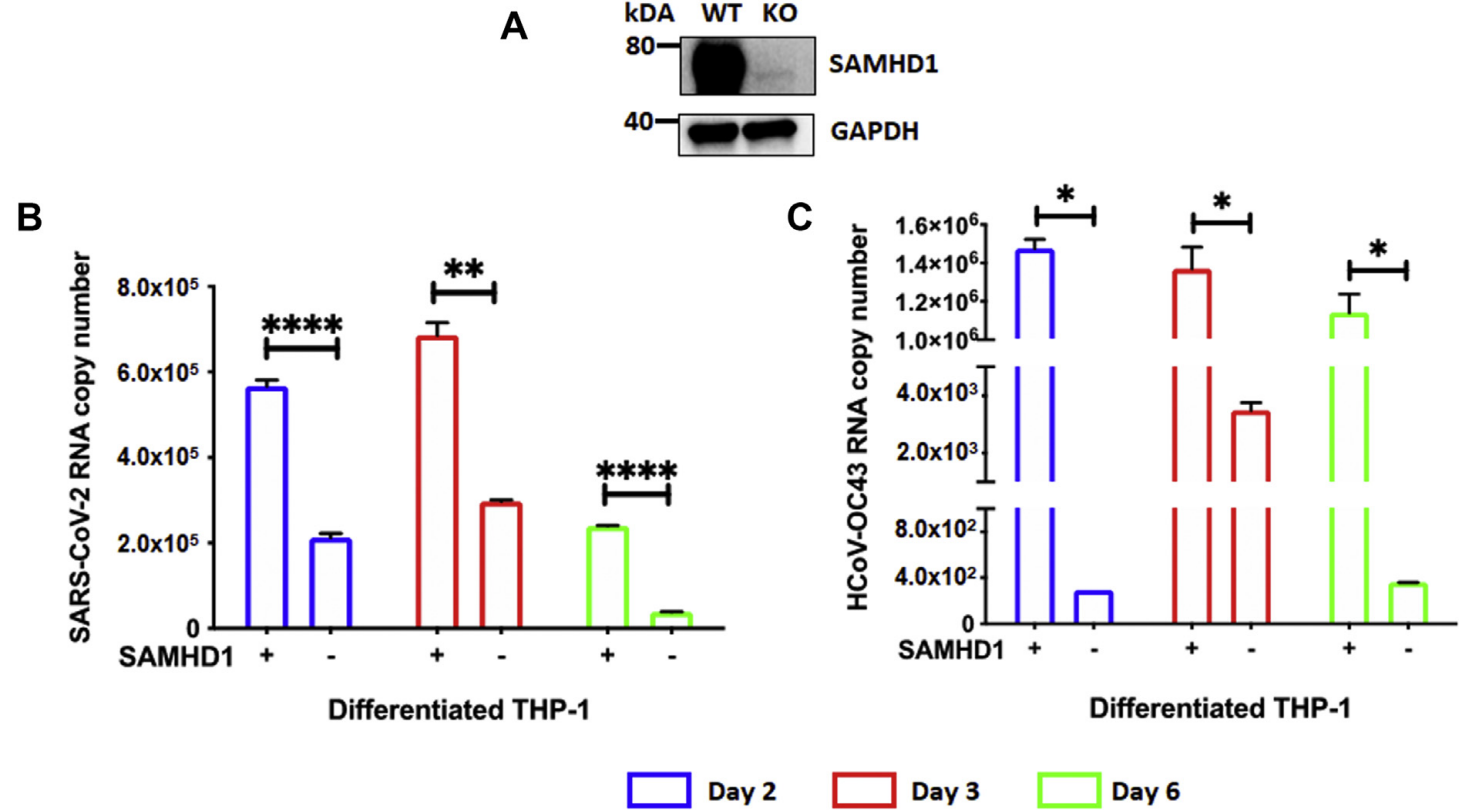
**SAMHD1 loss downregulates SARS-CoV-2 and HCoV-OC43 RNA levels in differentiated THP-1 macrophages.***A*, SAMHD1 expression in PMA-differentiated SAMHD1 WT and KO THP-1 macrophages was confirmed *via* Western blot using antihuman SAMHD1 antibody. GAPDH was used as a loading control. Separately, using 96-well plates, differentiated SAMHD1 WT and KO THP-1 cells were infected with either (*B*) SARS CoV-2 or (*C*) HCoV-OC43 at MOI 0.1 in triplicates, and intracellular RNAs were isolated on days 2 (*blue*), 3 (*red*), and 6 (*green*) postinfection for qRT–PCR analyses. The data are presented as means of triplicates, and the standard deviations from the means are represented as error bars. HCoV-OC43, human coronavirus OC43; MOI, multiplicity of infection; PMA, phorbol 12-myristate 13-acetate; qRT, quantitative RT; SAMHD1, sterile alpha motif and histidine–aspartate domain–containing protein 1; SARS-CoV-2, severe acute respiratory syndrome coronavirus.

Macrophages are important target cells during SARS-CoV-2 infection and crucial for the subsequent development of viral pathogenesis in COVID-19 patients (39). Although macrophages are permissive to SARS-CoV-2 infection, they do not support the rapid and productive viral replication and new viral protein syntheses that are observed in Vero cells, a cell line that are commonly used for antiviral drug screening purposes (Fig. S1; (40, 41)). Albeit the limited nature of SARS-CoV-2 replication capability in macrophages, the high SAMHD1 expression in terminally differentiated macrophages provided us with a useful tool to further evaluate the effect of SAMHD1 loss on the virus activity in these myeloid cells. Hence, using SAMHD1 WT- and KO-differentiated/nondividing THP-1 macrophages, we determined and compared the intracellular viral RNA copy numbers of SARS-CoV-2 and HCoV-OC43 postinfection. SAMHD1 expression in SAMHD1 WT- and KO-differentiated THP-1 cells (42) was validated by Western blot (Fig. 2*A*). Following differentiation with phorbol 12-myristate 13-acetate for 3 days, THP-1 macrophages were infected with SARS-CoV-2 (MOI = 0.1) and HCoV-OC43 (MOI = 0.1). Intracellular RNA samples were collected on days 2, 3, and 6 postinfection, and the viral RNA copy numbers were determined in order to evaluate the effects of SAMHD1 loss on SARS-CoV-2 and HCoV-OC43. As shown in Figure 2, *B* and *C*, we observed that the SAMHD1 KO-differentiated THP-1 cells generated lower intracellular RNA levels of both SARS-CoV-2 (Fig. 2*B*; 57–84% reduction) and HCoV-OC43 (Fig. 2*C*; 99% reduction) across all time points (Fig. 2, *B* and *C*), compared with the SAMHD1 WT THP-1 cells. Although SARS-CoV-2 and HCoV-OC43 RNA levels increased from day 2 to day 3 postinfection (Fig. 2, *B* and *C*), possibly because of the existing virus replication machinery elements from initial virus particles, the lack of new viral protein syntheses within the infected cells (Fig. S1*C*) may result in the observed gradual decline in RNA copy numbers during the later time point on day 6 (Fig. 2, *B* and *C*). Furthermore, the decrease in viral RNA copy numbers was not because of changes in host cell viability as SARS-CoV-2-infected WT and KO THP-1 cells remained highly viable across the different time points postinfection (Fig. S2). Overall, the data in Figure 2 support that the genetic loss of *SAMHD1* also suppresses SARS-CoV-2 and HCoV-OC43 RNA levels in differentiated THP-1 macrophages.

### Lentivirus viral protein X protein suppresses SARS-CoV-2 RNA levels in primary human monocyte–derived macrophages

**Figure 3:**
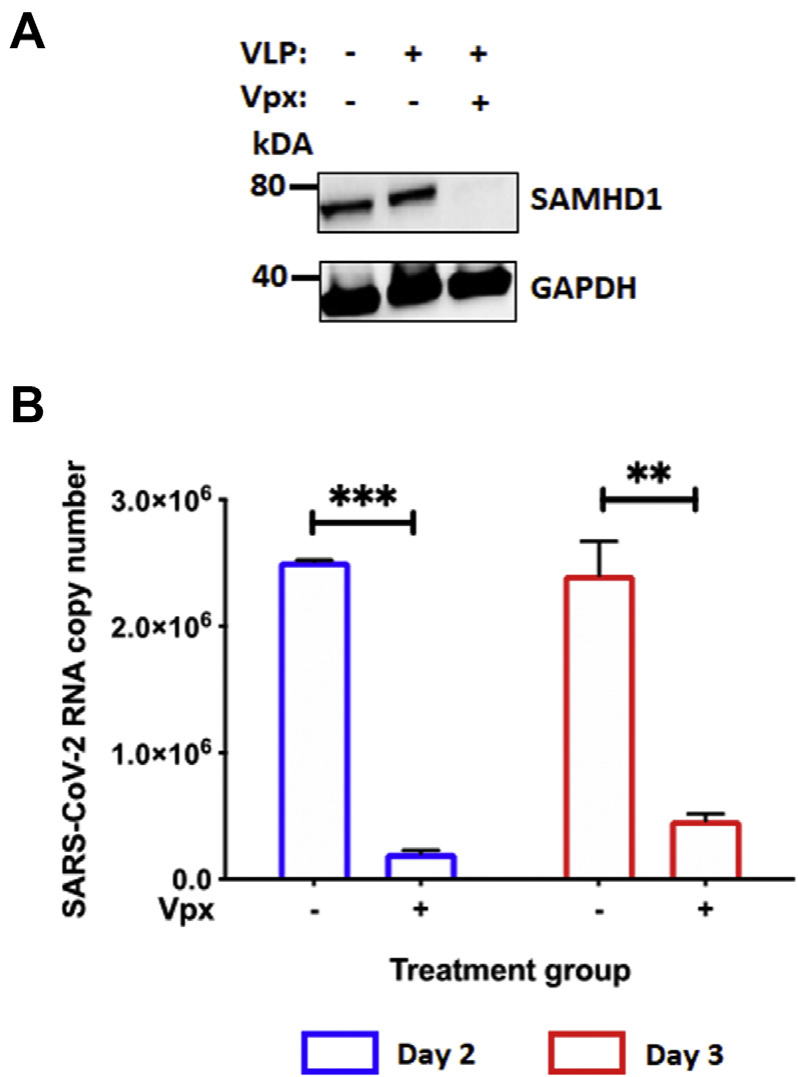
**Vpx treatment suppresses SARS-CoV-2 replication in primary human monocyte–derived macrophages (MDMs).***A*, SAMHD1 expression in primary human MDMs treated with either VLP Vpx (−) or Vpx (+) was confirmed *via* Western blot using antihuman SAMHD1 antibody. GAPDH was used as the loading control. MDMs were prepared from GM-CSF-mediated 7-day differentiation of human primary monocytes pooled from five healthy donors. *B*, separately, using 96-well plates, primary MDMs were treated with VLP Vpx (−) or Vpx (+) for 12 h in triplicates, before the cells were infected with SARS-CoV-2 (MOI = 0.1). On days 2 (*blue*) and 3 (*red*) postinfection, intracellular RNAs were isolated from the infected MDMs for SARS-CoV-2 RNA copy numbers quantification *via* qRT–PCR. The data are presented as means of triplicates, and the standard deviations from the means are represented as error bars. GM-CSF, granulocyte–macrophage colony-stimulating factor; MOI, multiplicity of infection; qRT, quantitative RT; SAMHD1, sterile alpha motif and histidine–aspartate domain–containing protein 1; SARS-CoV-2, severe acute respiratory syndrome coronavirus 2; VLP, virus-like particle; Vpx, viral protein X.

As SARS-CoV-2 and HCoV-OC43 were found to be effectively suppressed by the loss of SAMHD1 in the macrophage-like cell line, differentiated THP-1 cells (Fig. 2), we further verified these observations using primary human monocyte–derived macrophages (MDMs). As HCoV-OC43 replication is strongly restricted in primary MDMs (43), we investigated only SARS-CoV-2 infection in MDMs. Host SAMHD1 is targeted for ubiquitin-mediated proteosomal degradation by lentivirus viral protein X (Vpx), which is expressed by HIV-2 and several simian immunodeficiency virus strains (44). Thus, SAMHD1 protein levels in primary MDMs can be downregulated by the treatment with Vpx-containing virus-like particles (VLPs) (45). In the present study, granulocyte–macrophage colony-stimulating factor–differentiated primary human MDMs, which were pretreated for 12 h with Vpx– or Vpx+ VLPs, were infected with SARS-CoV-2 in triplicates. Subsequently, intracellular RNAs were extracted on days 2 and 3 postinfection. SAMHD1 protein levels in primary MDMs treated with VLP Vpx– or Vpx+ were validated by Western blot (Fig. 3*A*). Relative to the VLP Vpx– treated cells, SAMHD1 degradation by VLP Vpx+ significantly downregulated SARS-CoV-2 intracellular RNA copy number on days 2 (91.7%) and 3 (80.6%) postinfection (Fig. 3*B*). The data in Figure 3 support that loss of SAMHD1 protein reduces SARS-CoV-2 viral RNA copy numbers in human primary MDMs as observed in both 293T cells (Fig. 1) and differentiated THP-1 cells (Fig. 2).

### STAT1 mRNA level and protein phosphorylation are upregulated in the absence of SAMHD1

**Figure 4:**
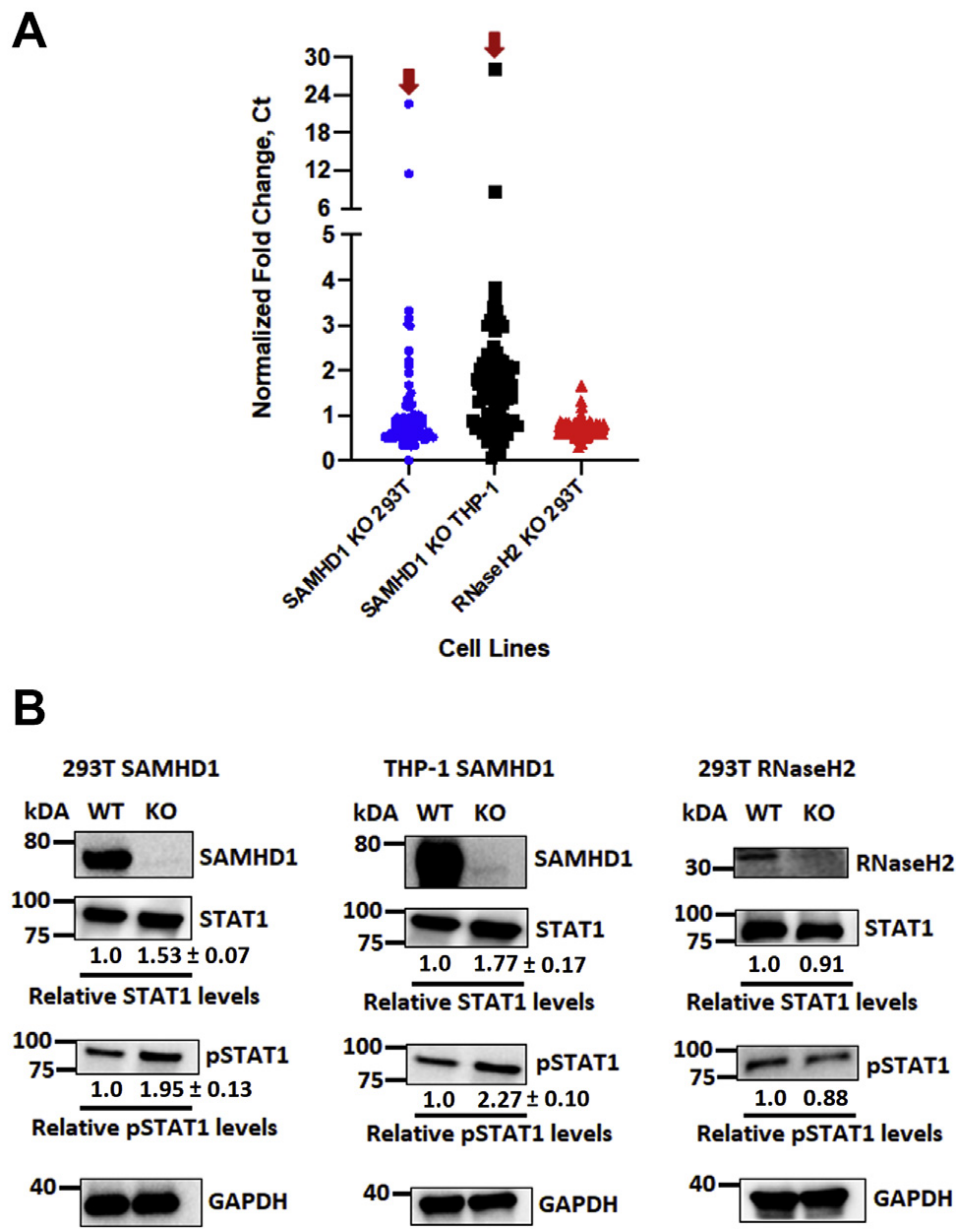
**Human interferon pathways genes and proteins are upregulated in the absence of SAMHD1.** Total intracellular RNA isolated from SAMHD1 WT and KO 293T and differentiated THP-1 macrophages as well as RNaseH2 WT and KO 293T cells was used for random cDNA fragment syntheses. The resulting cDNA samples were utilized to evaluate human interferon gene expression using the TaqMan Array Human Interferon Pathway, Fast 96-well (Thermo Fisher Scientific). *A*, the qPCR Ct values of each gene in SAMHD1 KO and RNaseH2 KO cells were compared and normalized with the WT cells and presented as normalized fold change computed using the Livak method (47). Four housekeeping genes embedded in the array were used for the signal normalization. *Red arrows* indicate STAT1 mRNA fold change in SAMHD1 KO 293T and differentiated THP-1 cells. Normalized fold changes of specific mRNAs of each cell line are presented in Figs. S3–S5. *B*, STAT1 and pSTAT1 protein expressions in each cell line, in the presence or the absence of SAMHD1 or RNaseH2 expression, were evaluated *via* Western blot using antihuman STAT1 and pSTAT1 antibodies. GAPDH was used as a loading control. The relative STAT1 and pSTAT1 protein levels were normalized with GAPDH, and the ratios between respective WT and KO cells were calculated. Relative changes in STAT1 and pSTAT1 protein expression in SAMHD1 KO cells were presented as means of triplicates ± standard deviations from the means. SAMHD1 Western blot data of 293T and differentiated THP-1 cells were from the same blots presented in Figures 1*A* and 2*A*. cDNA, complementary DNA; pSTAT1, phosphorylated form of STAT1; qPCR, quantitative PCR; SAMHD1, sterile alpha motif and histidine–aspartate domain–containing protein 1; STAT1, signal transducer and activator of transcription 1.

In addition to its dNTPase activity, SAMHD1 was revealed as an important negative regulatory factor of the human innate immune response (34). In fact, the *SAMHD1* gene is one of the genes that, if mutated, results in the development of the autoimmune disorder, AGS (27, 46). SAMHD1 has been shown to suppress IFN-1 production by reducing the phosphorylation of the NF-B inhibitory protein (IκBα), thus suppressing the activation of NF-κB (34). Our present study investigated SAMHD1-associated changes in mRNA expression levels of different IFN pathway genes in 293T and differentiated THP-1 cells to elucidate the underlying factor contributing to the inhibitory effects of SAMHD1 on SARS-CoV-2 and HCoV-OC43 replication (Figure 1, Figure 2, Figure 3). For this, we conducted a quantitative RT–PCR (qRT–PCR)-based RNA array analysis with cellular RNAs extracted from an equal number of uninfected SAMHD1 WT and KO 293T as well as differentiated THP-1 cells in duplicates. In particular, we employed the human IFN pathway array system to identify changes in specific IFN pathway genes, in the presence or the absence of SAMHD1 expression. The qRT–PCR Ct value normalization was conducted with four different housekeeping genes as recommended by the array manufacturer. The qRT–PCR Ct values of each gene from SAMHD1 KO cells were compared with those from the WT cells and presented as normalized fold change computed using the Livak method (47). As shown in Figs. S3*A* and S4*A*, all type 1 IFNs/IFN receptors detected in the array were upregulated in SAMHD1 KO cells, whereas higher mRNA levels were also observed for gene expression regulatory elements such as histones and various transcription/translation factors of IFN-activated genes, relative to SAMHD1 WT cells. A previous publication also similarly reported the upregulation of different IFN and inflammation signaling pathways when SAMHD1 expression was absent in KO THP-1 cells (34). While the mRNA levels of most IFN pathway genes (guanine nucleotide exchange factors, phosphoinositide 3-kinase/Akt/mammalian target of rapamycin, mitogen-activated protein kinase, and JAK–STAT signaling pathways) were upregulated in the absence of SAMHD1 (Figs. S3*B* and S4*B*), elevation of STAT1 mRNA in both SAMHD1 KO 293T and differentiated THP-1 cells was the most prominent (see *red arrows* in Figs. 4*A*, S3 and S4). Hence, by performing Western blot analyses, we further determined whether the molecular changes of STAT1 mRNA expression in the absence of SAMHD1 would be translated into increased protein production of STAT1 as well as its phosphorylated and active form (pSTAT1) in the host cells. As shown in Figure 4*B*, our Western blot findings demonstrated that both total STAT1 and pSTAT1 levels were upregulated at varying levels when SAMHD1 expression was abolished in both 293T and differentiated THP-1 cells. Our findings showed that 53% and 77% enhancement in STAT1 protein expression levels was detected in SAMHD1 KO 293T and differentiated THP-1 cells, respectively, whereas pSTAT1 expressions were increased by 95% and 127% for respective cell lines when these protein levels were normalized with GAPDH (Fig. 4*B*).

Separately, we evaluated the effects of genetic loss by CRISPR–Cas9 KO of another AGS-associated gene, *RNaseH2*, on the different IFN pathway gene expression as well as STAT1 and pSTAT1 protein production levels in 293T cells. However, in contrast with our findings from SAMHD1 KO 293T cells, the absence of RNaseH2 did not result in any change in the tested parameters (Figs. 4, *A* and *B* and S5), which could explain the minimal to no change in SARS-CoV-2 and HCoV-OC43 replication observed in RNaseH2 KO 293T cells relative to the WT cells (Fig. S6). RNaseH2 KO in 293T cells failed to induce innate immunity gene expression and suppress the replication of these two beta-coronaviruses likely because RNaseH2 induces the innate immune response through the cyclic GMP–AMP synthase–stimulator of interferon genes pathway, which is absent in 293T cells (48, 49).

### JAK inhibitor treatment overrides anti-SARS-CoV-2 effects of SAMHD1 KO in host cells

**Figure 5:**
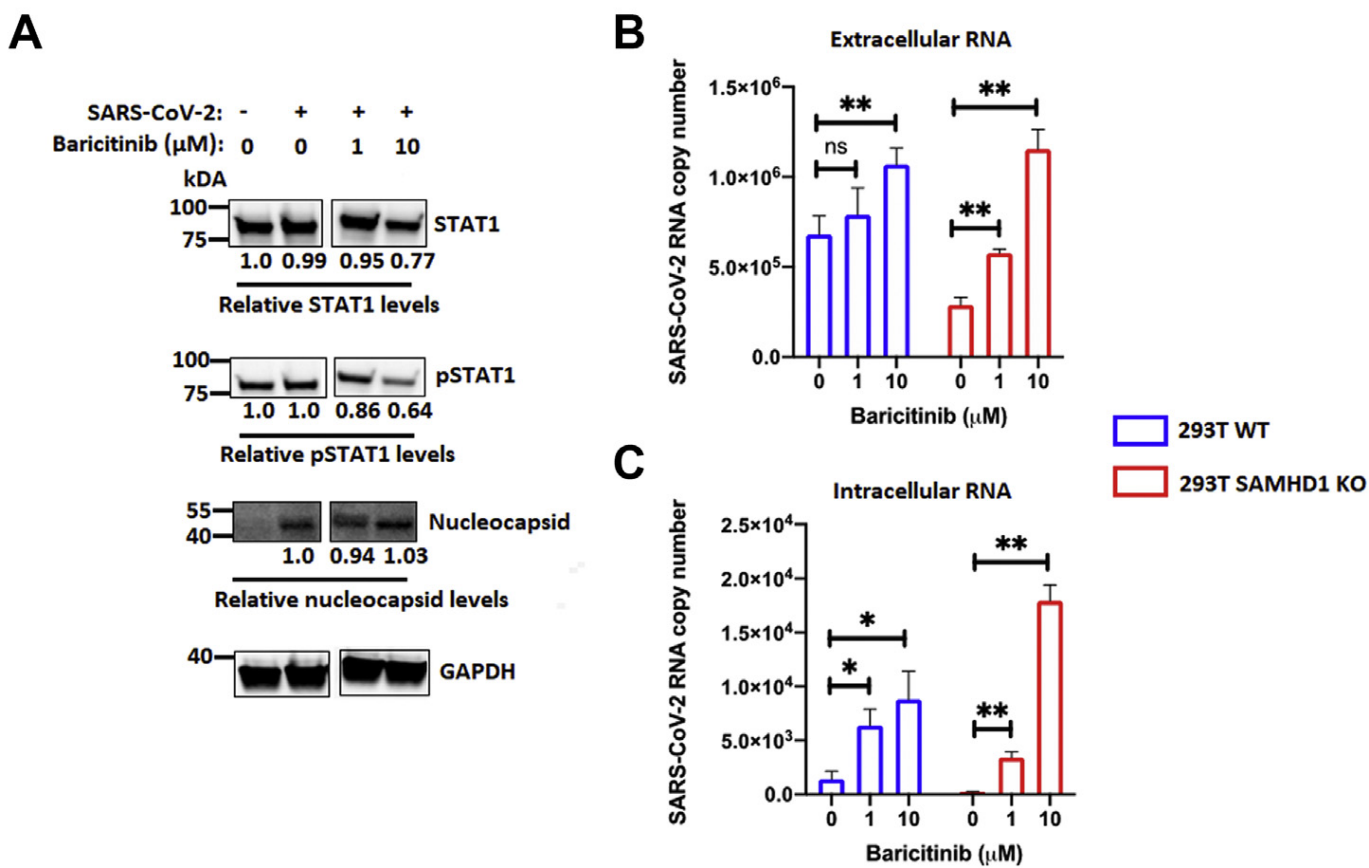
**Baricitinib treatment overrides the anti-SARS-CoV-2 effects of SAMHD1 KO.***A*, STAT1 and pSTAT1 protein expressions in SAMHD1 WT 293T cells following treatment with different concentrations of baricitinib as indicated were evaluated *via* Western blots using antihuman STAT1 and pSTAT1 antibodies. SARS-CoV-2 nucleocapsid and human GAPDH were presented as infection and loading controls, respectively. The relative STAT1, pSTAT1, and nucleocapsid protein levels were normalized with GAPDH, and the ratios of each protein between respective treatment groups with the mock-infected or mock-treated cells were calculated. *B*, SAMHD1 WT (*blue*) and KO (*red*) 293T cells were pretreated with different concentrations of baricitinib as indicated for 1 h, and the cells were infected with SARS-CoV-2 (MOI = 0.1). On day 2 postinfection, extracellular (*B*) and intracellular (*C*) RNA in the media and cells, respectively, were isolated for SARS-CoV-2 RNA copy number quantification *via* qRT–PCR. The data are presented as means of triplicates, and the standard deviations from the means are represented as error bars. MOI, multiplicity of infection; pSTAT1, phosphorylated form of STAT1; qRT, quantitative RT; SAMHD1, sterile alpha motif and histidine–aspartate domain–containing protein 1; SARS-CoV-2, severe acute respiratory syndrome coronavirus 2; STAT1, signal transducer and activator of transcription 1.

Baricitinib is an anti-inflammatory drug that selectively inhibits JAK1–JAK2 signaling, hence restricting the downstream STAT1 phosphorylation and activation (50). It has been utilized in clinical settings for patients suffering from autoimmune diseases such as systemic lupus erythematosus and rheumatoid arthritis (51, 52). In fact, the FDA has recently approved the use of baricitinib as an individual drug treatment for hospitalized SARS-CoV-2-infected adults and children aged 2 years or older, who are on supplemental oxygen support (https://www.fda.gov/media/143822/download). In this study, we investigated whether treatment with baricitinib would affect SARS-CoV-2 replication and eliminate the anti-SARS-CoV-2 activity resulting from elevated IFN responses in the absence of SAMHD1. Hence, we pretreated SAMHD1 WT and KO 293T cells with baricitinib 1 h prior to SARS-CoV-2 infection. While our low treatment group (1 μM) did not result in much change in protein expression levels of STAT1 and pSTAT1, both proteins were effectively downregulated in the high treatment group (10 μM) (Fig. 5*A*). Subsequently, we observed a significant dose-dependent elevation of extracellular viral RNA released into the media of SARS-CoV-2-infected SAMHD1 WT and KO 293T cells subjected to both treatment groups of baricitinib (Fig. 5*B*). While we observed relatively weak induction in extracellular viral RNA copy numbers from cells treated with 1 μM baricitinib (Fig. 5*B*), the downregulation of STAT1 and pSTAT1 protein expression in the high treatment group (10 μM) (Fig. 5*A*) resulted in comparable extracellular viral yields between both SAMHD1 WT and KO 293T cells (Fig. 5*B*). When we analyzed the intracellular RNA samples, even 1 μM of baricitinib treatment was sufficient to significantly enhance the viral RNA copy number in SAMHD1 KO cells, in comparison to the no treatment control where little intracellular viral RNA was detected. The data in Figure 5 suggest that baricitinib, which negatively regulates STAT1 and pSTAT1, overrides the elevated innate immunity associated with SAMHD1 loss. This results in enhancement of SARS-CoV-2 replication regardless of SAMHD1 status in the target cells.

## Discussion

In response to a viral infection, the first line of defense in the host immune system is the innate immune response, which is characterized by the massive production of IFNs and other proinflammatory cytokines. Host cell detection of pathogen-associated molecular patterns from the invading virus by the pattern recognition receptors will activate multiple signaling cascades, eventually leading to upregulated transcription of various ISGs. The resulting ISGs inhibit viral replication by either directly interrupting the virus life cycle or stimulating the production of antiviral factors by the infected and neighboring bystander cells (53, 54, 55, 56). However, disrupted IFN production during SARS-CoV-2 infection results in a high inflammation–low antiviral response imbalance as observed in majority of severe stage COVID-19 patients (2). Similar to the earlier SARS-CoV-1, which led to little to no production of IFN following infection of macrophages (57, 58), SARS-CoV-2 infections also result in low IFN inductions (2, 59). This is mainly because of viral proteins encoded by the coronaviruses, which allow them to evade detection and the induction of a host antiviral response. Upon pathway activation by viral nucleic acids, mitochondrial antiviral-signaling protein phosphorylates IRF3 and IRF7, which then migrate to the nucleus to induce ISG expression and IFN-1 production (60). SARS-CoV-1 and SARS-CoV-2 suppress host innate immunity by targeting mitochondrial antiviral-signaling protein *via* their accessory protein ORF9b (61, 62). Furthermore, using a luciferase reporter assay, expression of other SARS-CoV-2 proteins, such as membrane (M), ORF3, ORF6, nonstructural protein 1 (NSP1), NSP3, NSP12, NSP13, and NSP14, significantly inhibited IFN-β promoter activation (63). Other crucial IFN signaling factors such as TANK-binding kinase and the ubiquitin ligase, RNF41, have close binding interactions with SARS-CoV-2 NSP13 and NSP15, respectively (61). These findings as a whole further support the idea that since SARS-CoV-2 harbors multiple viral factors that can counteract host INF responses, this virus could be highly sensitive toward high antiviral IFN environments.

In spite of the roles of IFNs in the host protective immunity from invading viruses, overexpression of IFNs is highly detrimental and could lead to the development of inflammatory disorders such as AGS. Abnormal elevation of IFN levels during early development is among the most important clinical phenotype observed in AGS patients, which results from mutational loss of functions in several proteins associated with nucleic acid metabolism, such as SAMHD1 (21, 24, 27). SAMHD1 suppresses the host innate immune response by inhibiting NF-κB and IFN pathway activation. The NF-κB signaling pathway was significantly upregulated in SAMHD1 KO THP-1 cells in comparison to that of the WT control cells, whereas SAMHD1 silencing in primary macrophages led to elevated gene expression of IFN-1 and proinflammatory factors such as interleukin 6 and tumor necrosis factor alpha (34). In the same study, Sendai virus infection and exogenous stimulation by lipopolysaccharides only resulted in enhanced NF-κB and other proinflammatory factors in the KO cells but not in the SAMHD1-expressing cell population.

Our present study showed that in the absence of IFN-negative regulators like SAMHD1, SARS-CoV-2 replication was effectively reduced in multiple human cell lines and primary cells (Figure 1, Figure 2, Figure 3), possibly because of elevated innate immune responses as indicated by the upregulation of various ISGs in SAMHD1 KO cells, STAT1 mRNA in particular (Figs. 4*A*, S3 and S4). The higher mRNA expression of STAT1 was subsequently found to translate into elevated levels of both total STAT1 and activated pSTAT1 proteins (Fig. 4*B*). The increases in both total and pSTAT1 levels in SAMHD1 KO cells suggest that the innate immune response associated with JAK–STAT signaling was actively induced within the SAMHD1 KO cells, which could lead to the suppression of SARS-CoV-2 replication. Interestingly, our array analysis also detected strong stimulation of the Rho guanine nucleotide exchange factor 5 (ARHGEF5) mRNA in SAMHD1 KO cells, albeit relatively weaker than that of STAT1 mRNA expression. The ARHGEF5, like any other Rho guanine nucleotide exchange factors, belongs to a family of cellular proteins that activate GTPases in response to infection or inflammatory stimuli and is involved in vital signaling of immune cell proliferation, migration to sites of infection, differentiation, and activation (64). This group of host proteins is crucial in the production of cytokines such as IFNs and has key roles in regulating cellular innate immunity (65).

Separately, ACE2, a key target cell receptor of SARS-CoV-2, was also reported to be an ISG (66). Indeed, we observed that the loss of SAMHD1 promoted higher *ACE2* gene expression in differentiated THP-1 cells (Fig. S7), which is consistent with the report that SAMHD1 suppresses IFN-mediated innate immunity (34). However, even with this enhanced *ACE2* gene expression, SARS-CoV-2 RNA levels were suppressed in SAMHD1 KO cells, suggesting that this antiviral effect induced by SAMHD1 loss occurs at postviral entry step(s). As SARS-CoV-2 and HCoV-OC43 replication do not involve the reverse transcription step that utilizes dNTPs as substrates (67), unlike HIV-1 (68), the observed effects following SAMHD1 loss are unlikely because of changes in nucleotide metabolism within the KO cells. Furthermore, the growth and viability of host cells were not negatively affected following SAMHD1 KO as previously reported (42). Hence, the decrease in viral RNA copy numbers detected in KO cells did not result from lower amount of viable host cells to support productive virus replication.

The JAK–STAT pathway is the main antiviral IFN signaling cascade, which leads to transcription and expression of a variety of ISG products. Following IFNs binding to IFN receptors, STATs are phosphorylated by activated JAKs or other tyrosine kinases and translocate to the cell nuclei to induce gene transcription. The importance of the JAK–STAT IFN signaling in restricting replication of different viruses has been observed with enteric viruses such as hepatitis E virus, rotavirus, and human norovirus (69, 70), whereas inhibition of the JAK–STAT pathway promotes replication of Hantaan virus (71). On the other hand, in addition to its function in antiviral IFN production, the role of JAK–STAT signaling in the release of a wide array of proinflammatory cytokines during SARS-CoV-2 infections has been the center of attention for various research groups across the globe. Currently, there are multiple ongoing clinical trials involving JAK inhibitors as part of the therapeutic regimens to manage hyperinflammatory conditions in infected individuals. For instance, a clinical trial (the Adaptive COVID-19 Treatment Trial 2) involving 67 trial sites from eight countries, reported that COVID-19 patients who have been treated with remdesivir in combination with baricitinib exhibit improved clinical outcomes as well as enhanced recovery rates, in comparison with individuals treated with remdesivir alone (19). A separate phase 3 clinical trial (COV-BARRIER) also showed that baricitinib treatment was associated with lower COVID-19-associated mortality among infected patients (20). Based on the promising safety profiles and effectivity of baricitinib in managing the disease progression among COVID-19 patients, the FDA has recently approved the use of baricitinib as an individual drug treatment for SARS-CoV-2-infected individuals (https://www.fda.gov/media/143822/download). Apart from baricitinib, a phase 2 clinical trial known as RuxCoFlam, involving a potent JAK1/2 inhibitor, ruxoltinib, was also initiated. In this trial, ruxoltinib was administered into stage 2 and stage 3 COVID-19 patients for 7 days. Primary outcome measures of that study showed that hospitalized COVID-19 patients administered with ruxolitinib exhibited marked decrease in inflammatory markers without showing vital signs of toxicity (72).

Furthermore, our study suggests that disruption of the JAK–STAT signaling pathway by JAK inhibitor, baricitinib, was found to result in elevation of SARS-CoV-2 replication regardless of the state of innate immunity in the host cells (Fig. 5, *B* and *C*). This was evident as the decrease of STAT1 and the activated pSTAT1 protein expression in 293T cells following baricitinib treatment (10 μM) (Fig. 5*A*) resulted in relatively similar extracellular viral yields from infected SAMHD1 WT and KO 293T cells (Fig. 5*B*). The elimination of the inhibitory effect on SARS-CoV-2 replication observed in the absence of the negative regulation of antiviral IFN responses by SAMHD1 (Figure 1, Figure 2, Figure 3), by baricitinib (Fig. 5, *B* and *C*), further supports the role of a functional innate immune response in regulating the virus replication.

However, as our tissue culture system involved the use of CRISPR–Cas9 gene KO of *SAMHD1*, the permanent genetic loss of this protein limited us from looking further into the kinetics of STAT1 phosphorylation and induction of various ISGs between the SAMHD1 WT and KO cell lines. Besides, 293T cells in general have not been the primary choice of cell lines used to study IFN response to viral infections, mainly because of the lack of several innate immunity signaling pathway components such as cyclic GMP–AMP synthase–stimulator of interferon genes (48, 49). In spite of that, studies have shown that this cell line still harbors basal levels of ISGs, which are inducible by exogenous interventions or specific gene modifications (73, 74). Hence, this has provided us with a suitable model in this study to evaluate the differences in SARS-CoV-2 and HCoV-OC43 replication within the weak and induced states of IFN expression between SAMHD1 WT and KO 293T cells, respectively. Furthermore, our study also investigated the effects of another AGS protein KO, RNaseH2, which could only be genetically silenced while maintaining cell viability in the event of the resulting DNA damage, in p53-expressing human cell lines such as 293T cells (75, 76, 77). Collectively, our *in vitro* tissue culture model systems suggest that genetic loss of *SAMHD1* could suppress coronavirus (SARS-CoV-2 and HCoV-OC43) replication in multiple cell types, and this could be due to the resulting enhancement in cellular innate immune response signaling and antiviral IFN production. Our findings further support the importance of IFNs as a crucial antiviral or a therapeutic option to inhibit SARS-CoV-2 in COVID-19 patients.

## Experimental procedures

### Cells

Primary human monocytes were isolated from peripheral blood mononuclear cells of four healthy donors, which were purchased from the New York Blood Service, using the MACS CD14 microbeads (Miltenyi Biotec) as described previously (78). The pooled monocytes from five donors were differentiated for 7 days into MDMs in the presence of 5 ng/ml human granulocyte–macrophage colony-stimulating factor (Miltenyi Biotec). Separately, 293T and THP-1 cells (42) transduced with LentiCRISPR empty vector control (SAMHD1 WT) or vector containing specific guide RNA targeting the *SAMHD1* gene (SAMHD1 KO) were cultured in Dulbecco's modified Eagle's medium (DMEM) and RPMI, respectively, which were supplemented with 10% fetal bovine serum (FBS), penicillin–streptomycin (100 U/ml), and puromycin (1 μg/ml) at 37 °C, 5% CO_2_. Separately, 293T cells that have been LentiCRISPR–Cas9 KO for *RNaseH2* gene expression (RNaseH2 KO) were cultured under the same conditions as SAMHD1 KO 293T cells. The monocytic THP-1 cells were differentiated into macrophage-like nondividing cells, *via* treatment with 100 ng/ml phorbol 12-myristate 13-acetate for 72 h. On the other hand, Huh-7 and Vero E6 cells (American Type Culture Collection) were grown and maintained in DMEM and minimal essential medium containing 10% FBS and penicillin–streptomycin (100 U/ml) at 37 °C, 5% CO_2_, respectively.

### VLPs

VLPs (Vpx −/+) were generated as described in our previous publication (79). In T225 tissue culture flasks, 293T cells cultured in DMEM containing 10% FBS and 100 U/ml penicillin–streptomycin were transfected with 40 μg pVpx– VLP or pVpx+ VLP (kindly provided by Dr Florence Margottin-Goguet and Dr Nathaniel Landau) as well as 20 μg pVSV-g in the presence of 1 mg/ml of polyethylenimine. After cellular debris was removed from supernatants collected on days 2 and 3 post-transfection *via* centrifugation at 1200 rpm for 7 min, the VLPs were concentrated by ultracentrifugation (22,000 rpm) at 4 °C for 2 h. The resulting pellets dissolved in Hank's balanced salt solution were flash-frozen with ethanol and stored in aliquots at −80 °C.

### SARS-CoV-2 and HCoV-OC43

SARS CoV-2 (catalog no.: NR-52281: USA-WA/2020) and HCoV-OC43 were obtained from BEI Resources and American Type Culture Collection, respectively. SARS CoV-2 was propagated in Vero cells, whereas HCoV-OC43 was cultured in Huh-7 cells. Both viruses were titrated using the 50% tissue culture infectious dose method. The virus stocks were aliquoted and stored at −80 °C until needed.

### Virus yield quantification by qRT–PCR

Intracellular and extracellular RNA were extracted from cell lysates and supernatants using the TRIZOL and TRIZOL LS reagents (Thermo Fisher Scientific), respectively, according to the manufacturer's protocol. The resulting RNA samples were used for one-step qRT–PCR analysis using the qScript XLT One-Step RT–qPCR ToughMix (QuantaBio), according to the parameters and instructions provided by the manufacturer. The primers and probe used for SARS CoV-2 RNA quantification were 2019-nCoV_N1-F (5′-GACCCCAAAATCAGCGAAAT-3′), 2019-nCoV_N1-R (TCTGGTTACTGCCAGTTGAATCTG-3′), and 2019-nCoV_N1-Probe (5′-FAM-ACCCCGCATTACGTTTGGTGGACC-BHQ1-3′), whereas HCoV-OC43 was detected using the forward primer (5′-ATGTTAGGCCGATAATTGAGGACTAT-3′), reverse primer (5′-AATGTAAAGATGGCCGCGTATT-3′), and probe (5′-6-FAM-CATACTCTG/ZEN/ACGGTCACAAT-3IABkFQ-3′). All primers and probes were purchased from Integrated DNA Technologies.

### Statistical analyses

Data analyses were performed using GraphPad Prism for Windows (version 8; GraphPad Software, Inc). Unpaired *t* tests were used to determine the significance of each reading relative to its respective control in each dataset. The results are presented as means ± SE, whereby only significant datasets were labeled as follow: *p* < 0.05 was indicated as ∗*p* < 0.01 was indicated as ∗∗*p* < 0.001 was indicated as ∗∗∗; and *p* < 0.0001 was indicated as ∗∗∗∗.

### Human IFN pathway gene expression analyses

Cellular RNA from SAMHD1 WT, KO 293T, and THP-1 cells as well as RNaseH2 WT and KO 293T cells was isolated using the RNeasy Mini Kit (Qiagen), according to the manufacturer's protocol. Different fragments of complementary DNA (cDNA) were generated from random primers *via* reverse transcription using the High-Capacity cDNA Reverse Transcription kit (Thermo Fisher Scientific). The cDNA synthesis thermal cycling conditions include 25 °C for 10 min, 37 °C for 2 h, and 85 °C for 5 min. About 50 ng of the resulting cDNA was added into each well of the TaqMan Array Human Interferon Pathway, Fast 96-well (Thermo Fisher Scientific), containing primer–probe mix of respective target genes, and the TaqMan Gene Expression Master Mix (Thermo Fisher Scientific). Expression levels of each gene were determined as Ct values *via* qPCR with thermal cycle parameters of 50 °C for 2 min, 95 °C for 10 min, and 40 cycles of 95 °C for 15 s and 60 °C for 1 min.

### Western blot

Respective cell lines were cultured to a density of 5 × 10^5^ cells per well in 12-well plates and lysed with cold radioimmunoprecipitation assay buffer in the presence of 1× Halt Protease and Phosphatase Inhibitor (Thermo Fisher Scientific). Collected cell lysates were spun down at 16,000*g* for 5 min to remove cell debris. The resulting lysates were denatured with Laemmli buffer (Bio-Rad) at 95 °C for 5 min and subjected to SDS-PAGE, before being transferred onto nitrocellulose membranes. Proteins of interest in this study were detected *via* primary antibodies specific for human SAMHD1 (catalog no.: 67820; Abcam), human STAT1 (catalog no.: 234400; Abcam), human pSTAT1 (catalog no.: 109461; Abcam), human RNaseH2 (catalog no.: 83943; Abcam), SARS-CoV-2 nucleocapsid (catalog no.: NUN-S47; ACROBiosystems), and human GAPDH (catalog no.: 2118; Cell Signaling). Secondary antibodies used in this study were antimouse (catalog no.: NA931V; Cytiva) and anti-rabbit (catalog no.: NA934V; Cytiva) antibodies. Following the addition of SuperSignal West Femto Maximum Sensitivity Substrate (Thermo Fisher Scientific), the blots were visualized using ChemiDoc Touch Imaging System (Bio-Rad).

## Data availability

All data presented in this study are contained within the article and available from authors upon request.

## Supporting information

This article contains supporting information (47).

## Conflict of interest

The authors declare that they have no conflicts of interest with the contents of this article.
