# Supplementary material for: Elimination of Aicardi–Goutières syndrome protein SAMHD1 activates cellular innate immunity and suppresses SARS-CoV-2 replication
Source: J Biol Chem. 2022 Jan 25;298(3):101635. doi: 10.1016/j.jbc.2022.101635 (PMC8786443; doi:10.1016/j.jbc.2022.101635)
Supplement: Supplemental Figures S1–S7 [file mmc1.docx]

**FIGURE S1**


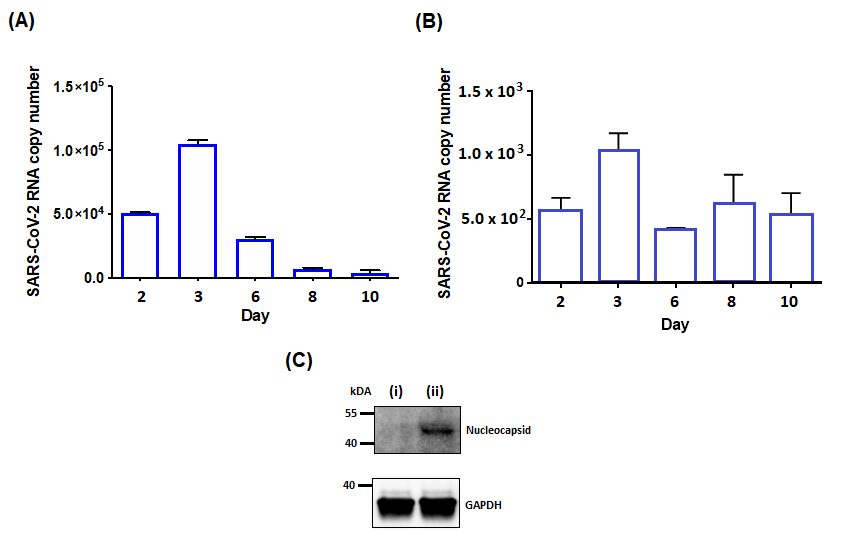


**Figure S1. SARS-CoV-2 exhibits abortive replication in macrophages.** Using 96-well plates, primary MDMs were infected with SARS-CoV-2 (MOI 0.1) in triplicates, and the (A) intracellular and (B) extracellular RNA samples were isolated on Days 2, 3, 6, 8 and 10 post-infection for qRT-PCR analyses of SARS-CoV-2 RNA copy numbers. All qRT-PCR data are presented as means of triplicates, and the standard deviations from the means are represented as error bars. (C) At 48 h post-infection, SARS-CoV-2 nucleocapsid protein expression in infected (i) primary macrophages and (ii) Vero cells were evaluated by western blot using anti-SARS-CoV-2 nucleocapsid antibody. GAPDH was used as a loading control.

**FIGURE S2**

**
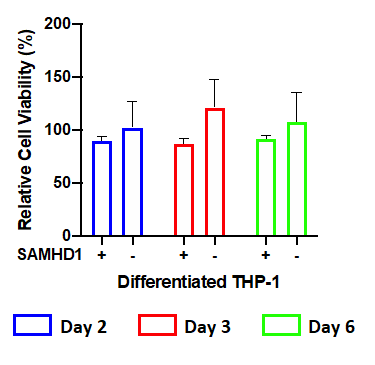
**

**Figure S2. Differentiated THP-1 macrophages remain highly viable during SARS-CoV-2 infection.** Using 96-well plates, SAMHD1 WT and KO differentiated THP-1 cells were infected with SARS-CoV-2 (MOI 0.1) in triplicates. On Days 2, 3 and 6 post-infection, MTS reagent (Promega) was added into each well and incubated for 1 h at 37°C. Optical density of each well was measured at 490 nm using a microplate reader. Cell viability of the infected cells were normalized with mock-infected control cells at respective time points. Data are presented as means of triplicates, and the standard deviations from the means are represented as error bars.

**FIGURE S3**


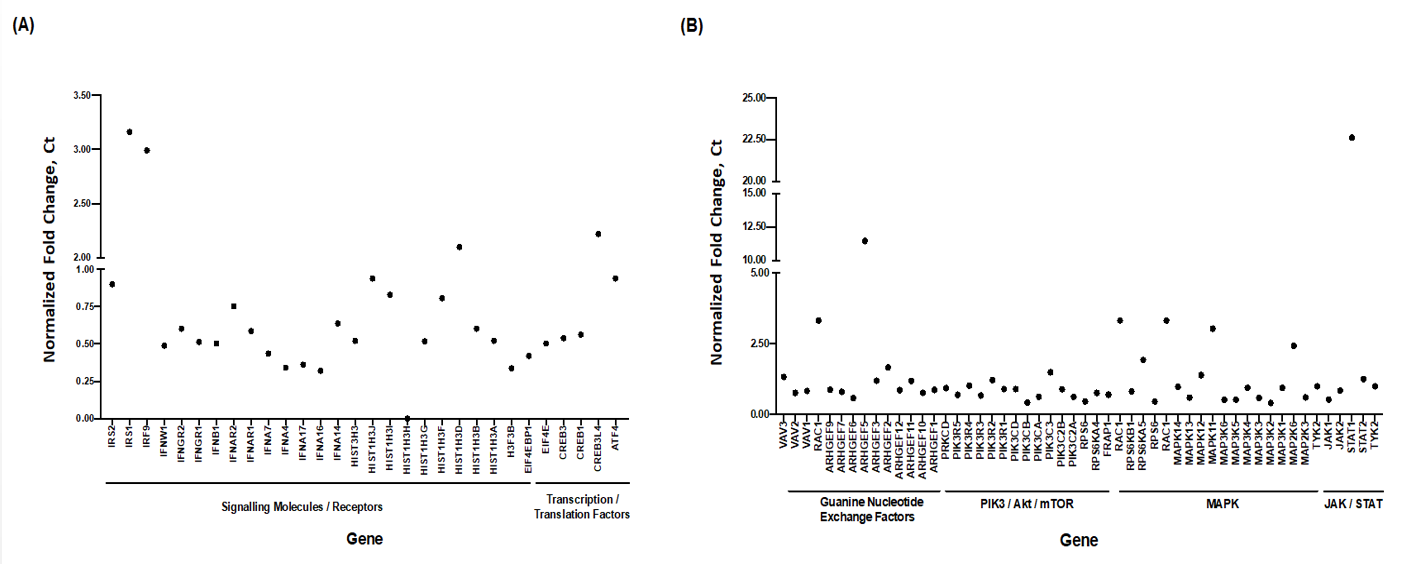


**Figure S3. Human interferon pathways genes are upregulated in SAMHD1 K/O 293T cells.**  Total intracellular RNA isolated from SAMHD1 WT and K/O 293T cells were used for random cDNA fragments syntheses. The resulting cDNA samples were utilized to evaluate human interferon genes expression using the TaqMan™ Array Human Interferon Pathway, Fast 96-well (Thermo Fisher Scientific). The qPCR Ct values of (A) IFNs, IFNRs, histones and transcription/translation factors, as well as (B) various IFN signaling pathways in SAMHD1 K/O 293T cells were compared and normalized with the WT cells, and presented as normalized fold change computed using the Livak method (47).

**FIGURE S4**


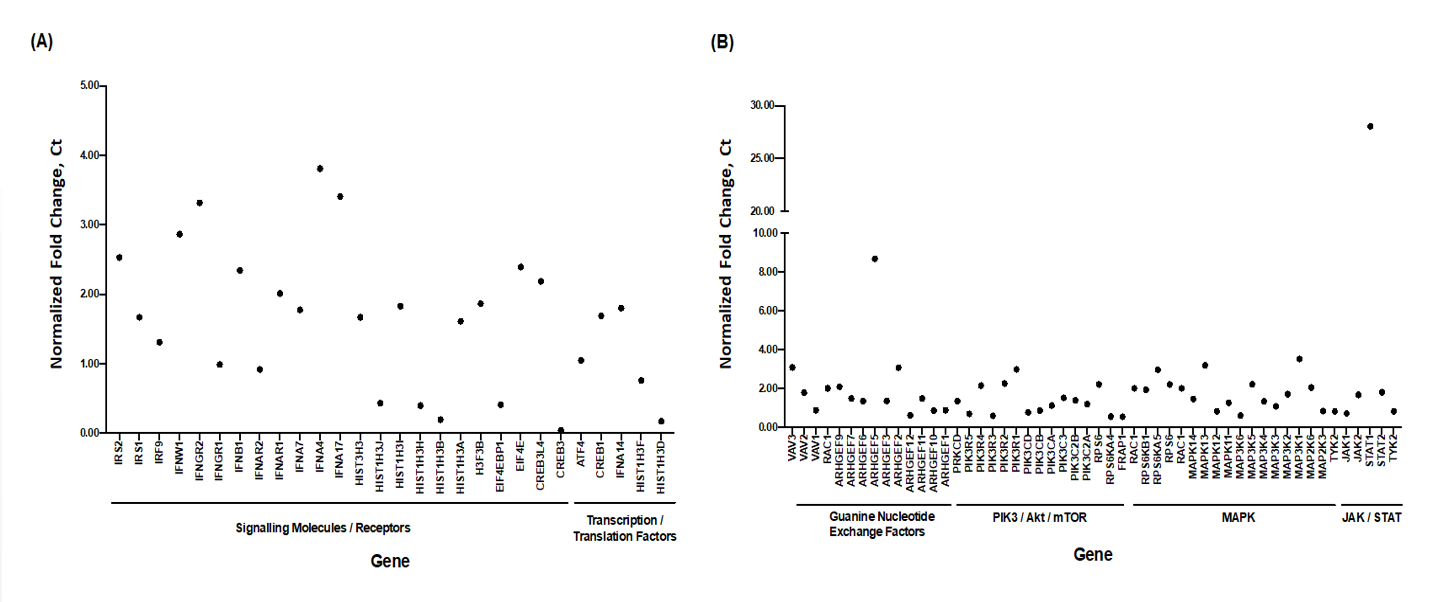


**Figure S4. Human interferon pathways genes are upregulated in SAMHD1 K/O differentiated THP-1 cells.**  Total intracellular RNA isolated from SAMHD1 WT and K/O differentiated THP-1 cells were used for random cDNA fragments syntheses. The resulting cDNA samples were utilized to evaluate human interferon genes expression using the TaqMan™ Array Human Interferon Pathway, Fast 96-well (Thermo Fisher Scientific). The qPCR Ct values of (A) IFNs, IFNRs, histones and transcription/translation factors, as well as (B) various IFN signaling pathways in SAMHD1 K/O differentiated THP-1 cells were compared and normalized with the WT cells, and presented as normalized fold change computed using the Livak method (47).

**FIGURE S5**


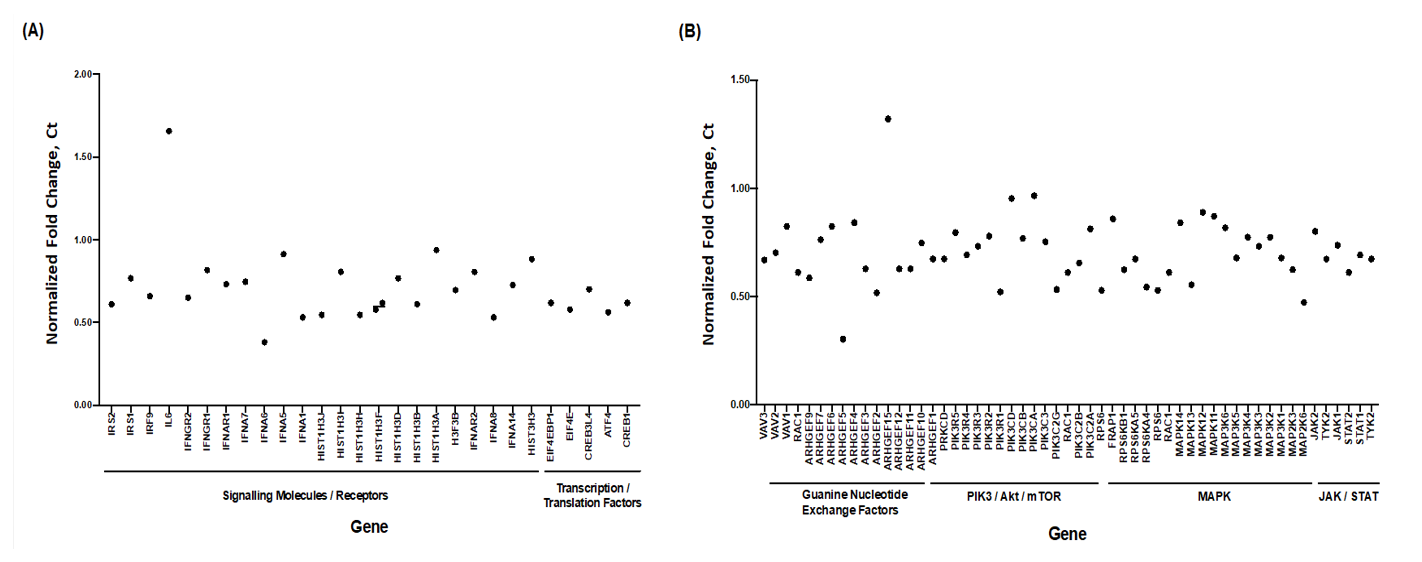


**Figure S5. Human interferon pathways genes are not affected by RNaseH2 loss in 293T cells.**  Total intracellular RNA isolated from RNaseH2 WT and K/O 293T cells were used for random cDNA fragments syntheses. The resulting cDNA samples were utilized to evaluate human interferon genes expression using the TaqMan™ Array Human Interferon Pathway, Fast 96-well (Thermo Fisher Scientific). The qPCR Ct values of (A) IFNs, IFNRs, histones and transcription/translation factors, as well as (B) various IFN signaling pathways in RNaseH2 K/O 293T cells were compared and normalized with the WT cells, and presented as normalized fold change computed using the Livak method (47).

**FIGURE S6**


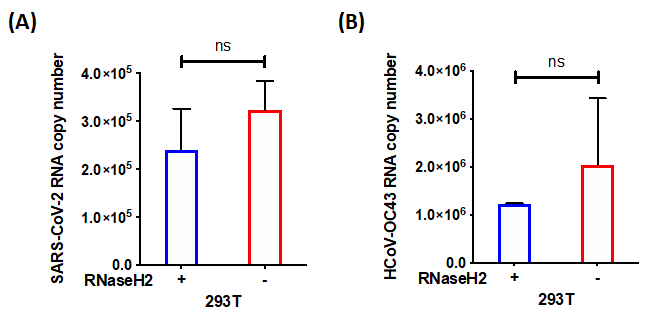


**Figure S6. RNaseH2 loss does not suppresses SARS-CoV-2 and HCoV-OC43 replication in 293T cells.** RNaseH2 protein expression in RNaseH2 WT and K/O 293T cells were verified by western blot using anti-human RNaseH2 antibody (Figure 4). RNaseH2 WT and K/O 293T cells were infected with either (A) SARS CoV-2 or (B) HCoV-OC43 at MOI 0.1 in triplicates, before extracellular viral RNA samples were isolated from the media on Day 2 post-infection for qRT-PCR analyses. The data are presented as means of triplicates, and the standard deviations from the means are represented as error.

**FIGURE S7**

**Figure S7. ACE2 gene expression levels in differentiated SAMHD1 +/- THP-1 and Vero E6 cells.** Total intracellular RNA of differentiated SAMHD1 WT (untreated; 48 h-treatment with VLP Vpx (+) or different types of IFNs-1 at 100 IU/ml) and K/O THP-1 cells, as well as Vero E6 cells were extracted using the TRIZOL RNA extraction method. The resulting RNA samples were subjected to ACE2 gene expression analyses via qRT-PCR. The data are presented as means of triplicates, and the standard deviations from the means are represented as error bars.
